# Supplementary material for: Hospitals’ Collaborations Strengthen Pandemic Preparedness: Lessons Learnt from COVID-19
Source: Healthcare (Basel). 2024 Jan 26;12(3):321. doi: 10.3390/healthcare12030321 (PMC10855495; doi:10.3390/healthcare12030321)
Supplement: Supplementary file 1 [file healthcare-12-00321-s001.zip › healthcare-2763586-supplementary.pdf]

Supplementary Materials S1: Good Reporting of a Mixed Methods Study (GRAMMS)

**Guideline**	**Section: page**
Describe the justification for using a mixed methods approach to the research question	Study design and population: p2<br><br>Strengths and limitations: p19
Describe the design in terms of the purpose, priority and sequence of methods	Study design and population: p2
Describe each method in terms of sampling, data collection and analysis	Study participants: p4<br><br>Data collection: p5-6<br><br>Data analysis: p6-7
Describe where integration has occurred, how it has occurred and who has participated in it	Data synthesis: p7
Describe any limitation of one method associated with the present of the other method	Discussion: p18 lines 24-32
Describe any insights gained from mixing or integrating methods	Discussion: p17 lines 25-30

O'Cathain A, Murphy E, Nicholl J. The quality of mixed methods studies in health services research. J Health Serv Res Policy. 2008;13(2):92-98

Supplementary Materials S2: Knowledge Questionnaires Pre and Post Training

Table S1: Score calculation for Infection Control, Nursing, PCR, and housekeeping quizzes among partner hospitals.

	Questions	Answer choices
**House Keeping quiz questions**	1-How does the transmission of COVID-19 occur?	**a. By droplet and contact**<br>b. By airborne transmission<br>c. By Droplet transmission<br>d. From food containers and contaminated packages
	2-The recommended time for alcohol-based solutions use is?	a. 60 seconds<br>b. 90 seconds<br>**c. 20-30 seconds**<br>d. 3 minutes
	3-Which of the following applies while wearing a surgical mask?	a. The surgical mask should cover both the nose and the mouth<br>b. Keep using the surgical mask even if damp<br>c. The front part of the mask should not be touched by hands<br>**d. a and c**
	4-What should we wear before entering the isolation room of a COVID-19 patient?	a. A gown and a surgical mask<br>b. A surgical mask only<br>c. Gown, surgical mask, and goggles/face shield<br>**d. Gown, surgical mask, googles/face shield, and gloves**
	5-How frequently should the high touch surfaces (door handles, chairs, etc.) be cleaned/disinfected?	a. Every 2 hours<br>b. Every 6 hours<br>c. Every 8 hours<br>**d. Twice per day**
	6-Which solutions should be used for disinfection after discharge of covid-19 patients?	a. Water with any cleaning detergent<br>b. No need to use any solution, close room for 24 hours<br>**c. Hydrogen peroxide or bleach for terminal disinfection**<br>d. Alcohol 70%

**Infection control quiz questions**	1-How does the transmission of COVID-19 occur?	**a. By droplet and contact**<br>b. By airborne transmission<br>c. By Droplet transmission<br>d. From food containers and contaminated packages
	2-The recommended time for alcohol-based solutions use is?	a. 60 seconds<br>b. 90 seconds<br>**c. 20-30 seconds**<br>d. 3 minutes
	3-Which of the following applies while wearing a surgical mask?	e. The surgical mask should cover both the nose and the mouth<br>f. Keep using the surgical mask even if damp<br>g. The front part of the mask should not be touched by hands<br>**h. a and c**
	4-What should we wear before entering the isolation room of a COVID-19 patient?	a. A gown and a surgical mask<br>b. A surgical mask only<br>c. Gown, surgical mask and goggles/face shield<br>**d. Gown, surgical mask, googles/face shield, and gloves**
	5-Which one of these is not an aerosol generating procedure?	a. Bronchoscopy<br>b. Cardiopulmonary resuscitation<br>**c. Taking vital signs for the COVID patient**<br>d. Intubation of the patient
**Nursing quiz questions**	1-How does the transmission of COVID-19 occur?	**a. By droplet and contact**<br>b. By airborne transmission<br>c. By Droplet transmission<br>d. From food containers and contaminated packages
	2-The recommended time for alcohol-based solutions use is?	a. 60 seconds<br>b. 90 seconds<br>**c. 20-30 seconds**<br>d. 3 minutes
	3-Which of the following doesn't apply while wearing a surgical mask?	**a. The surgical mask should cover both the nose and the mouth**

		b. Hand hygiene should be performed only after disposing the mask<br>c. The surgical mask should be disposed before leaving patient's room<br>d. Remove the mask by touching the front part of the mask with your hands
	4-What should we wear before entering the isolation room of a COVID-19 patient?	a. A gown and a surgical mask<br>b. A surgical mask only<br>c. Gown, surgical mask and goggles/face shield<br>**d. Gown, surgical mask, googles/face shield, and gloves**
	5-Which one of these is not an aerosol generating procedure?	a. Bronchoscopy<br>b. Cardiopulmonary resuscitation<br>**c. Taking vital signs for the COVID patient**<br>d. Intubation of the patient
	6-What should you do before leaving the room of a patient after performing AGP?	**a. Remove gloves and protective gown**<br>b. Remove gloves/protective gown/mask and wash your hands<br>c. Remove all PPE then wash hands<br>d. Leave the room then remove PPE and wash hands
	7-When performing high flow nasal oxygen therapy (HFNC), which of the bellow applies?	a. Place the patient in a negative pressure or in a well-ventilated room<br>b. The health care worker must wear N95 mask<br>c. Place a surgical facemask on patient to cover the HFNC<br>**d. All of the above**
	8-Who should you notify first when a patient is suspected to have Covid-19?	a. Hospital administrator<br>**b. Infection control team and Attending physician**<br>c. Media<br>d. Ministry of public health
	9-Which steps should be followed when a suspected covid-19 patient is identified?	a. Provide the patient with a surgical face mask

		b. Place all suspected cases in one small waiting room<br>c. Keep at least 2 meters distance between suspected patients when waiting<br>**d. a and c**
	10-Which of the below applies for a deceased covid-19 patient?	a. Full PPE should be worn before getting in contact with the body<br>b. Treat the body as per hospital procedures and religious rituals<br>c. The body should be placed in one impermeable bag<br>**d. a and b**
**PCR sampling quiz questions**	1-What is the preferred choice for diagnostic testing for COVID-19?	a. Viral culture<br>b. Antibody testing<br>**c. RT-PCR**<br>d. Chest X-Ray
	2-Which of the following should be worn when taking a nasopharyngeal swab on a patient?	a. Gloves and N95 mask<br>b. Long sleeve gown, surgical mask and gloves<br>**c. Long sleeve gown, N95 mask, gloves and goggles/face shield**<br>d. N95 mask, gloves and goggles/face shield
	3-When performing PCR test, the examiner should?	a. Stand facing the mouth of the patient<br>b. Take the swab only from one nostril<br>c. Place swab in tube and give to patient<br>**d. Put the swab in a special tube and transport to the laboratory**
	4-What should we wear before entering the isolation room of a COVID-19 patient?	a. No differences at all<br>**b. No difference in technique, the only difference is the estimate distance from nose to ear**<br>c. For pediatrics and neonates, swab from one nostril only is enough

***For each correct answer (bolded) a score of 1 is given, while a score of 0 is given for all other incorrect choices.**

Supplementary Materials S3: Training Evaluation Questionnaires

Table S2: Trainer evaluation score

Trainer evaluation questions:	Answer	Score per answer
Was the trainer knowledgeable about the training topic?	Very poor<br>Poor<br>Good<br>Very good	**1**<br>**2**<br>**3**<br>**4**
Did the trainer encourage participation during the training?	Very poor<br>Poor<br>Good<br>Very good	**1**<br>**2**<br>**3**<br>**4**
Did the trainer do a good and organized preparation?	Very poor<br>Poor<br>Good<br>Very good	**1**<br>**2**<br>**3**<br>**4**
Did the trainer communicate clearly and effectively?	Very poor<br>Poor<br>Good<br>Very good	**1**<br>**2**<br>**3**<br>**4**
Were your questions clearly addressed?	Very poor<br>Poor<br>Good<br>Very good	**1**<br>**2**<br>**3**<br>**4**
Maximum achievable score	**20**	

Table S3: Content evaluation score

Content evaluation questions:	Answer	Score per answer
Was the content of the training clear and easily understood?	Very poor<br>Poor<br>Good<br>Very good	**1**<br>**2**<br>**3**<br>**4**
Was the content of the training organized properly?	Very poor<br>Poor<br>Good<br>Very good	**1**<br>**2**<br>**3**<br>**4**
Did the training meet your expectations/needs?	Very poor<br>Poor<br>Good<br>Very good	**1**<br>**2**<br>**3**<br>**4**
Did your knowledge/skills increase as a result of the training?	Very poor<br>Poor	**1**<br>**2**

	Good<br>Very good	**3**<br>**4**
Will you be able to apply what you learnt from the training in your job?	Very poor<br>Poor<br>Good<br>Very good	**1**<br>**2**<br>**3**<br>**4**
How do you evaluate the training overall?	Very poor<br>Poor<br>Good<br>Very good	**1**<br>**2**<br>**3**<br>**4**
Maximum achievable score	**24**	

Supplementary Materials S4: Hospital Preparedness Checklist

Checklist Preparedness: COVID 19

General Information 2
Section I. Structure for Planning and Decision Making 3
Section II. Development of Written COVID-19 Plan 4
Section III. Elements of a General COVID-19 Plan 6
Section IV. Facility communication 7
A. Internal communication / External communication 8
B. Data protection 8
Section V. Surge Capacity 9
Section VI. Consumables and Durable Medical Equipment and Supplies 11
Section VII. Human Resources 13
Section VIII. Identification and Management of Ill Patients 15
Section IX. Moving Patients in the Facility 17
Section X. Patient Placement 18
Section XI. Visitor Access 20
Section XII. Continuity of Essential Healthcare Services 21
Section XIII. Occupational Health 22
Section XIV. Education and Training 23
Section XV. Infection Control and Prevention 25
Section XVI. Laboratory Services 27

Section XVII. Waste Management 28
Section XVIII. Environmental Cleaning Element 28
Section XIX. Postmortem Care 29
Section XX. Essential Support Services 29
Contributing Experts 29
References 30

General Information:

Evaluation date:

Name of the hospital:

City:

Number of Beds:

Number of Beds Intensive Care Unit (ICU):

Microbiology laboratory: Yes /No

ICU beds for adults:
- With Isolation:
- With negative pressure:
- With neutral pressure:

ICU beds for pediatrics (if applicable):
- With Isolation:
- With negative pressure:
- With neutral pressure:

ICU beds for neonatology (if applicable):
- With Isolation:
- With negative pressure:
- With neutral pressure:

Total number of invasive mechanical ventilators:
Total number of non-invasive ventilators:

Name of evaluators:

Section SI: Structure for Planning and Decision Making

Structure for Planning and Decision Making	Completed	In-Progress	Not Started	Not applicable
COVID-19 planning has been incorporated into disaster planning and exercises for the hospital.				
The hospital assigned a committee responsible of COVID-19 preparedness planning.				
The established COVID-19 committee consists of members from various medical disciplines. (If it applies, attach the list of names, titles and contact information of each member)				
The responsibilities of the Chair and each member of the committee are well described.				
The COVID-19 committee shares information regarding COVID-19 and pandemic plans with local, regional, and international planning groups.				
All facility coordinators and leadership have reviewed the guidelines for COVID-19.				
Back-up staffs for directors and focal points to guarantee the continuity of decision-making and resource management in any situation are appointed.				
The place where COVID-19 preparedness planning committee members meet is identified.				
A well-equipped COVID-19 Operation or Call Center is prepared and ready for any emergency response at the level of the hospital.				
The process of keeping updated documentation and informing staff where to find information is available and accessible by all staff.				

Section SII: Development of Written COVID-19 Plan

Development of a written COVID-19 Plan	Completed	In-progress	Not Started	Not applicable
A written COVID-19 preparedness plan is available at the facility and accessible to all staff. (If applicable, insert location: ______________________________)				
The plan includes the following elements:<br>a. The name of the persons responsible of putting the plan into action				

b. The place where the plan will be implemented<br>c. The assignment of staff to implement the plan				
The responsibilities of each staff and department within the organization are well described in the plan.				
The responsibilities of backup personnel are well described in the plan.				
Back-up personnel are well trained on response objectives, priorities, policies and ready for implementation.				
Staff sick-leave / vacation policies are identified described in the plan.				
The processes of staff recruitment, rotation and training along with the budget needed have been identified.				
The plan describes ways of expanding hospital in-patient capacity as a result of increase in demand during COVID-19 pandemic.				
The hospital identifies its own gaps in coordination with MoPH and neighboring hospitals. Those gaps are described in the plan.				
The plan includes the policies and criteria for admitting, discharging and prioritizing patients based on the hospital capacity.				
The plan describes security rules that ensure the safety of patients, staff, and visitors.				
The plan describes alternative suppliers in case of main suppliers' shortages.				
The hospital assigned a person responsible for monitoring public health advisories and continuous update of the committee.				
A protocol was developed to detect and manage potential cases using telemedicine. The protocol should include guide on criteria for detecting a possible case, the diagnostics needed, infection control measures, as well as notifying respective public health agencies including the MoPH.				

Section SIII: <u>Elements of a General COVID-19 Plan</u>

Elements of a General COVID-19 Plan	Completed	In-Progress	Not Started	Not applicable

The elements of the plan are shared with all hospital staff, healthcare, and non-healthcare workers.				
The plan elements include transparent facts and policies regarding the role of the hospital during COVID-19 pandemic, the availability and use of resources, and safety measures within the facility.				
Policies and procedures of hospital data protection are included in the plan and shared with all staff.				
The procedures of reporting COVID-19 cases are identified in the plan.				
The safety and follow-up procedures related to hospital staff in direct contact with COVID-19 suspected cases are included in the plan.				
The plan includes plan related to bed-distancing and cohorting among patients during COVID-19 pandemic.				
The policies of moving and transporting patients are included in the plan. (cited in managing patients)				
Visitors' policies are included in the plan and shared with patients and their families. (cited in visitors policy)				

Section SIV: <u>Facility Communications</u>

Facility Communications	Completed	In-progress	Not Started	Not applicable
Safety workshops to train all hospital staffs on safety measures and importance of hygienic procedures are performed.				
All hospital staffs are trained on how to work PPEs and how to decrease the risk of transmittance of COVID-19 among hospital patients.				
All healthcare workers are informed about the protocols related to the use of medications in COVID-19 treatment.				
Cleaning personnel and janitors are trained on how to clean areas suspected of having COVID-19 infection.				
Hand hygiene and waste management procedures are shared with all hospital workers.				
All hospital workers are informed about sick leaves/personal leaves policy.				

Communication among facility staff is implemented in various ways (e.g. shared documents, door/wall signs, organized workshops, etc.)				
Brochures and booklets regarding COVID-19 related information are prepared and shared with patients and their family members.				
The hospital assigned staff responsible of updating the MoPH regarding COVID-19 pandemic suspected and positive cases.				
The hospital assigned staff responsible of planning discussions and implementation within the facility during COVID-19 pandemic.				
The hospital has in place a procedure to collect feedback or questions from healthcare workers as well as incident reports.				
All the internal protocols, communication lines and standard operating procedures are easily and centrally accessible for all workers; all staff know where to locate them				

A. <u>Internal communication / External communication</u>

Internal Communication/ External Communication	Completed	In-progress	Not Started	Not applicable
A person has been assigned responsibility for communications with staff, patients, and their families regarding the status and impact of COVID-19 in the facility. Plans and responsibilities for communication with patients and their family members have been developed. Primary (Name, Title, Contact Information): ____________________ Backup (Name, Title, Contact Information): ____________________				
An external communication plan has been established, including a mechanism to ensure that communication with the media and the public are checked for consistency and approved before released.				
Staff members have been informed of the external communication plan.				
A core communication team plus backups has been appointed. They coordinate external				

communications. The spokesperson/s and his/her/their backups have been appointed.				
A referral mechanism at the national level and related communication mechanisms has been established.				

B. Data Protection

Data Protection	Completed	In-progress	Not Started	Not applicable
There are mechanisms in place to ensure data protection in accordance with the legislation.				
All staffs have been reminded of data protection rules.				
Mechanisms for tele-triage (e.g. phone, email, smartphone apps, telemedicine) conform to data protection rules.				

Section SV: Surge Capacity

Surge Capacity	Completed	In-progress	Not Started	Not applicable
Surge capacity plans include strategies to help increase hospital bed capacity.				
Surge capacity plans include strategies for maximizing number of staff available for direct patient care.				
The maximum facility capacity, including the maximal number of ICU beds and mechanical ventilators (along with the required human resource capacities and supply capacity) has been calculated.				
There is a system to monitor bed occupancy (including the number of patients in isolation), the number of rooms used for isolation, and the number of rooms that can be potentially used for isolation.				
The numbers of patients in isolation that will, once the number reaches a certain threshold, trigger the progressive conversion of normal rooms to isolation rooms; the capacity for cohorting patients of the same disease has been calculated.				
The number and location of potential beds to be re-assigned as isolation rooms and a plan to re-				

allocate the non-isolated patients to other rooms has been established.				
All staff members are aware of the triggers and procedures to convert normal rooms to isolation rooms.				
Surge capacity plans include strategies to use in emergency departments to mitigate surge and accommodate additional patients. Strategies such as alternate triage sites, use of telemedicine, and call centers may be considered to reduce surge on the facility.				
Facility space has been identified that could be adapted for use as expanded inpatient beds and this information has been provided to local authorities.				
Plans are in place to increase physical bed capacity (staffed beds), including the equipment, trained personnel and pharmaceuticals needed to treat a patient with COVID-19 (e.g., ventilators, oxygen).				
Ethical issues concerning how decisions will be made in the event healthcare services must be prioritized and allocated (e.g., decisions based on probability of survival) have been discussed.				
A procedure has been developed for communicating changes in hospital status to health authorities and the public.				
Additional areas to be transformed into waiting rooms have been identified; the threshold number of patients that would trigger the use of these areas has been estimated.				
If possible, access to separate toilets and drinking water faucets is available for patients in the waiting and emergency rooms.				
An adequate amount of PPE of all sizes is available for healthcare workers and cleaning personnel.				

Section SVI: <u>Consumables and Durable Medical Equipment and Supplies</u>

Consumables and Durable Medical Equipment and Supplies	Completed	In-progress	Not Started	Not applicable
Estimates have been made of the quantities of essential patient care materials and equipment (e.g., intravenous pumps and ventilators,				

pharmaceuticals) and personal protective equipment (e.g., facemasks, respirators, gowns, gloves, eye protection, and hand hygiene products), that would be needed during at least an eight-week outbreak.				
Estimates have been shared with local, regional groups to better plan stockpiling agreements.				
A plan has been developed to address likely supply shortages (e.g., personal protective equipment), including strategies for using normal and alternative channels for procuring needed resources and strategies for conserving PPE				
A strategy has been developed for how priorities would be made in the event there is a need to allocate limited patient care equipment, pharmaceuticals, and other resources.				
A process is developed to track and report available medical supplies including supplies of facemasks, respirators (if available) , gowns, gloves, and eye protection (i.e., face shield or goggles).				
Additional physical space within the hospital was identified for the storage and stockpiling of additional supplies.				
A process is in place to ensure that the facility provides supplies and materials necessary to adhere to recommended infection prevention and control practices including:<br>a. Alcohol-based hand sanitizer for hand hygiene is available in every patient room (ideally both inside and outside of the room) and other patient care and common areas.<br>b. Sinks are well-stocked with soap and paper towels for hand washing.<br>c. Signs are posted immediately outside of patient rooms indicating appropriate IPC precautions and required personal protective equipment (PPE).<br>d. Tissues and facemasks for persons with respiratory symptoms to use near				

entrances and in common areas, with no-touch receptacles for disposal.<br>e. PPE is available immediately outside of the patient room and in other areas where patient care is provided.<br>f. Trash disposal bins are positioned near the exit inside each patient room to make it easy for staff to discard PPE after removal, prior to exiting the room, or before providing care for another patient in the same room.<br>g. EPA-registered hospital-grade disinfections or other products with label claims against human coronaviruses are used for frequent cleaning of high-touch surfaces and shared patient care equipment.				
The facility has a contingency plan that includes engaging their health department and healthcare coalition when they experience (or anticipate experiencing) supply shortages.				
There is a mechanism in place to ensure that equipment is in perfect working order and can be quickly replaced whenever necessary				
Adequate filters adapted for prevention for SARS-CoV2 and other bacteria are available for the respirators in use, stocked and accessible.				

Section SVII: <u>Human Resources</u>

Human Resources	Completed	In-progress	Not Started	Not applicable
Estimates have been made of staff absenteeism in advance and monitored continuously.				
For each unit or service, the minimum number of health-care workers and other hospital staff needed to ensure the sufficient operation of the unit or service is identified.				
Staff needs by unit or service are prioritized and personnel are distributed accordingly.				

The surge capacity of healthcare workers for triage, ER, ICU, laboratory, and the units where the patients will be placed has been assessed.				
The surge capacity of non-healthcare workers (e.g. administration, cleaning personnel, etc.) has been assessed.				
A mechanism for the recruitment, training and quick provision of all necessary administration needs as well as equipment for new staff on short notice is in place, and the budget is available and allocated.				
Ward staff are familiarized to work in high-demand areas (e.g. infectious disease wards, emergency and intensive care units) to support surge.				
A plan is in place to avoid burnout among healthcare and non-healthcare workers; a maximum number of working hours will be ensured, workloads will be equally distributed, minimum rest times between shifts have been determined, as have been breaks during regular work shifts; a contact point has been appointed who can be addressed if there are problems.				
The availability of the services of multidisciplinary psycho-social support teams for the families of staff and patients, including social workers, counselors, interpreters and clergymen is ensured.				
Reassigning staff at high risk for complications of COVID-19 acute respiratory infection is considered.				
There is a security team in place to ensure the safety of patients, staff and visitors and key supplies if needed.				
A security plan is in place to ensure safety and provide guidance for security incidents; this includes escorting personnel, patients or visitors if necessary; staff has been informed of the security plan.				
Possible security risks have been identified; threshold events that trigger additional resources or support from local authorities have been established.				

Section SVIII: <u>Identification and Management of Ill Patients</u>

Identification and Management of III Patients	Completed	In-progress	Not Started	Not applicable
Specifically-trained healthcare personnel have been assigned responsibility to implement triage, early recognition, and source control (isolating patients with suspected COVID-19).				
Ensure that health-care workers have a high level of clinical suspicion.				
The hospital has an updated process for triage (e.g., initial patient evaluation) and admission of patients during an outbreak of COVID-19 that includes the following:<br>a. Plans to post visual alerts (signs, posters) at entrances and in strategic places providing instruction on hand hygiene, respiratory hygiene, and cough etiquette that is language, format (i.e., prepared for individuals with visual, hearing or other disabilities) and reading-level appropriate.<br>b. Supplies will be made available (tissues, no-touch waste receptacles, alcohol-based hand sanitizer).<br>c. Facemasks will be available at triage for patients with respiratory symptoms.<br>d. Training of personnel on appropriate processes (e.g., questions to ask and actions to take) to rapidly identify and isolate suspect COVID-19 cases.<br>e. Ensure the application of standard, and droplet precautions at all times.<br>f. Designate an exclusive waiting and examination area for individuals presenting with respiratory symptoms and/or fever. Ideally patients would be at least 6 feet (2 m) apart in waiting areas.<br>g. Consider establishing additional areas for triage of patients on presentation at the hospital, possibly outside the hospital (medical tents).				
A tele-triage system to triage patients before they arrive at the hospital is in place: phone/email/telemedicine services are in place for possible cases; these services can also be used to coordinate the arrival of patients at the hospital if required.				

The population has been informed about the tele-triage services of the hospital because they were informed about these services through several channels.				
Criteria for prioritizing admission of patients to those in most critical need have been established.				
A process is in place following identification of a suspect COVID-19 case to include:<br>a. Immediate notification of facility leadership/infection control.<br>b. Notification of local or state health department soon after arrival.<br>c. A method to specifically track admissions and discharges of patients with COVID-19.				
Provide patient care following national and international guidelines. Ensure that all staffs are aware of the national and international guidelines for case management.				
Admission criteria and triage logistics (e.g., location, routes of entry/exit) are communicated to the relevant hospital personnel.				
Procedures are in place for the cleaning of common areas and equipment in these triage areas.				
Specific guidelines have been established for management of confirmed / suspected cases requiring surgical interventions / OBGYN care / interventional / invasive procedures.				

Section SIX: <u>Moving Patients in the Facility</u>

Moving Patients in the Facility	Completed	In-progress	Not Started	Not applicable
The movement of patients within the healthcare facility is limited to performing essential procedures.				
A surgical mask is worn by the isolated patient when he/she is moved inside the healthcare facility.				
The best routes for moving patients within the healthcare facility have been established; staff members have been informed.				
All healthcare workers preparing, moving, and receiving patients are aware of the conditions of these patients and have been trained in all				

relevant procedures, e.g. where to find PPE and how to use it.				

Section SX: <u>Patient Placement</u>

Patient Placement	Completed	In-progress	Not Started	Not applicable
The capacity of isolation beds and ICU beds in the hospital has been assessed.				
If the hospital has rooms with negative pressure, the maximal number of patients that can be hosted in each room according to the manufacturer has been determined.				
The maximal capacity for the isolation of patients has been estimated:<br>a. Maximum number of rooms that can be converted into isolation rooms (if there is increased need) has been calculated.<br>b. Maximum number of patients that can be cohorted in isolation rooms and number of potential isolation rooms has been calculated.<br>c. A plan is in place that indicates the criteria that would trigger the transformation of normal rooms into isolation rooms and also the order in which this process would be carried out; this includes a plan to re-allocate patients, facilitate their rapid discharge as soon as their clinical status allows for it, or treat patients at home.				
The staff know the plan and have been trained accordingly, e.g. the know how to use PPE, are familiar with protocols and new tasks that they may be assigned to them, etc.				
Airborne infection isolation (AII) rooms have been tested and certified for their effectiveness (within the timeframe indicated by local regulations).				
PPE for aerosol-generating procedures are available in sufficient numbers and sizes so they can be used in the isolation rooms when appropriate.				
Only a limited number of staff members are authorized to access the isolation rooms; they have been trained accordingly. Staff members who have access to isolation rooms are tracked				

and records are kept. A record of all staff members who have access to isolation rooms is kept so that all staff movements can be tracked.				
Staff members with access to isolation rooms should be limited to reduce the possibility of transmission among other patients.				
Ensure the availability of oxygen and means of respiratory support, as well as sufficient sedation for intubated patients.				
Oxygen masks and nasal canulae should be single-use.				
OR department has at least one room with negative pressure.				
Plan for COVID-19 dialysis patients is prepared (isolated dialysis rooms with negative or neutral pressure)				

Section SXI: Visitor Access

Visitor Access	Completed	In-progress	Not Started	Not applicable
Plans for visitor access and movement within the facility have been reviewed to limit their movement and avoid gathering.				
The hospital has plans and materials developed to post signs at the entrances to the facility instructing visitors not to visit if they have fever or symptoms of a respiratory infection.				
Visitors' temperature is checked at the entrance.				
Visitors should wear surgical masks.				
Limit visitors to those essential for COVID-19 patient support.				
Maintain a record of all persons entering COVID-19 rooms.				
If visitors are allowed to enter the room of a confirmed or suspected COVID-19 patient, the facility will:<br>a. Enact a policy defining what PPE should be used by visitors.<br>b. Provide instruction to visitors before they enter a patient room, on hand hygiene, limiting surfaces touched, and use of PPE according to current facility policy.				

c. A trained healthcare worker is available to check the correct donning and doffing of PPE.				

Section SXII: Continuity of Essential Healthcare Services

Continuity of Essential Healthcare Services	Completed	In-progress	Not Started	Not applicable
Plans include strategies for maintaining the hospital's core missions and continuing to care for patients with chronic diseases (e.g., hemodialysis and infusion services), women giving birth, emergency services, and other types of required non-COVID-19 care.				
Identify the resources (human resources and logistics) needed to ensure the continuity of the identified essential hospital services.				
Criteria have been developed for determining when to cancel elective admissions and surgeries.				
Plans for initiating and expanding use of call centers and telemedicine to be able to serve patients without face to face contact. These plans include communicating with patients about how to access the call line or telemedicine services.				
Adapt admission and discharge criteria and prioritize patients and clinical interventions according to available treatment capacity and demand.				

Section SXIII: Occupational Health

Occupational Health	Completed	In-progress	Not Started	Not applicable
The facility has employee sick leave policies that are non-punitive, flexible, and consistent with public health policies that allow ill healthcare personnel (HCP) to stay home.				
The facility follows the local/state public health authority's policies and procedures for monitoring and managing HCP with potential for exposure to COVID-19, including ensuring that HCP have ready access, including via telephone, to medical consultation.				
The facility instructs all staff including contractors, volunteers and students to regularly				

monitor themselves for fever and symptoms of COVID-19, as a part of routine practice.				
The facility has a process to conduct symptom and temperature checks prior to the start of any shift of asymptomatic, exposed HCP that are not work restricted.				
The facility has a process to identify and manage HCP with fever and symptoms of COVID-19. Symptomatic HCP should contact a provider for guidance and not present to the workplace, unless instructed.				
The hospital has a plan for monitoring and assigning work restrictions for ill and exposed HCP.				
The hospital has a respiratory protection program that includes medical evaluation, training, and fit testing of employees.				
The hospital has a process for auditing adherence to recommended PPE use by HCP.				

Section SXIV: <u>Education and Training</u>

Education and Training	Completed	In-progress	Not Started	Not applicable
A plan for the general and specific training of all personnel is in place to help understand the implications of, and basic prevention and control measures for, COVID-19:<br>a. Plans for regular training updates to refresh concepts<br>b. Training for new personnel before they arrive or as soon as they arrive<br>c. To provide education for family members of patients				
All documents and procedures:<br>a. Are easily and centrally accessible<br>b. Staff has been informed where to find them				
A person or team is responsible designated with responsibility for coordinating education and training on COVID-19 (e.g., identifies and facilitates access to available programs, maintains a record of personnel attendance). (Insert name(s), title(s), and contact information.)<br>(Name, Title, Contact Information):				

(Name, Title, Contact Information):				
Training/information materials have been developed for education and job-specific training, in particular for:<br>a. Hand and respiratory hygiene<br>b. Who should use PPE: why, when and how<br>c. Internal and external communication lines and rules (both to receive and provide information)<br>d. Data protection with regard to patients<br>e. Triage procedures<br>f. Case definitions<br>g. Notification of cases<br>h. Placement and movement of patients in isolation and visitors' access<br>i. How to properly clean and disinfect environmental surfaces and equipment<br>j. Sick-leave policy and what to do if staff members show symptoms<br>k. Security plan<br>l. Where to find the documents and training material				
All staff, have been informed and trained on the topics mentioned above (double checklist), as required.<br>a. Healthcare workers<br>b. Non-healthcare workers				
Healthcare workers have been trained to minimize the specific risks related to the management of suspected or confirmed COVID-19 patients.				
Non-healthcare workers have been trained to minimize the specific risks related to their jobs, in particular the cleaning of areas occupied by a suspected or confirmed COVID-19 patient.				
Healthcare workers and cleaning personnel have been trained in putting on ('donning') and taking off ('doffing') PPE.				
Staff members who would have to wear PPE know the documents outlining the procedures.				
Language and reading-level appropriate materials have been identified to supplement and support education to patients, and family				

members of patients and a plan is in place for obtaining these materials.				

Section SXV: <u>Infection Control and Prevention</u>

Infection Control and Prevention	Completed	In-progress	Not Started	Not applicable
Standard precautions are applied on all patients by HCW (Hand hygiene, PPE when needed, mask on patients with respiratory symptoms…etc.)				
Droplets and contact precautions are implemented for suspected or confirmed COVID-19 cases				
Airborne precautions are implemented for aerosol-generating procedures (tracheal intubation, non-invasive ventilation, tracheotomy, cardiopulmonary resuscitation, manual ventilation before intubation, bronchoscopy, collection of nasopharyngeal swap/aspirate and autopsy). ventilation, collection of nasopharyngeal swab/aspirate and autopsy)				
Ventilated single rooms are available for isolation				
Single-used or disposable equipment is used; shared equipment (stethoscopes, BP cuffs) are cleaned/disinfected between patients				
Procedure for cleaning/disinfection of environmental surfaces is available and followed				
Hospital approved disinfectants are **adequate**				
Procedures for cleaning and disinfection of ambulances are available				
Where possible, a team of HCWs is designated to care exclusively for suspected/confirmed cases				
Ensure that staff (HCW, cleaning personnel) receive training on standard, contact, droplets, and airborne precautions (including correct use of PPE, donning and doffing, masks tested for fitting, hand hygiene, respiratory hygiene, etc.)				
Ensure that adequate PPE (facemasks, N95, gloves, gowns, eye protection) is **available** in different sizes and easily **accessible** to staff				

The need for PPE has been estimated				
Use designated portable X-ray equipment and/or other designated diagnostic equipment. If transport is required, use predetermined transport routes to minimize exposure for staff, other patients, and visitors, and have the patient use a medical mask if tolerable or reinforce respiratory hygiene. Ensure that HCWs who are transporting patients perform hand hygiene and wear appropriate PPE. Notify the area receiving the patient of any necessary precautions as early as possible before the patient's arrival.				
Visitors are limited and apply droplet and contact precautions.				
Laboratory specimens, laundry, food service utensils, and medical waste are safely managed				
Alcohol-based hand sanitizers are **adequate** and available especially in waiting rooms, triage rooms, examination rooms, and areas for the removal of PPE				
Soap and paper hand-towels are available in sufficient quantities next to all sinks (both in toilets and next to all hand wash sinks)				
A procedure to check and refill the supplies is established and working.				

Section SXVI: <u>Laboratory Services</u>

Laboratory Services	Completed	In-progress	Not Started	Not applicable
Basic laboratory testing is available.				
Essential supplies and resources are ensured .				
Laboratory personnel and their back-up are identified along with their services.				
Diagnostic laboratories are well equipped with sufficient amounts of reagents and supplies to ensure diagnostic tests of COVID-19.				
Staff are trained to handle COVID-19 specimen sampling while respecting infection control precautions (Swabs, Sputum, BAL …).				
A plan with criteria for identification and training of staff for sample collection, storage, specimen transportation or shipment for the identification, confirmation, and monitoring of COVID-19 should be in place in respect to infection control precautions national and				

international transport regulations and requirements.				

Section SXVII: Waste Management

Waste Management	Completed	In-progress	Not Started	Not applicable
The number of bins available on site can cover larger volumes of waste.				
Waiting and triage areas are equipped with no-touch bins to dispose of tissues used by patients.				
The facility can manage the increased amount of infectious waste or outsources its waste management. If yes describe how.				

Section SXVIII: Environmental Cleaning Element

Environmental Cleaning Element	Completed	In-progress	Not Started	Not applicable
A procedure has been established for the cleaning of the rooms on a regular basis and when required; cleaning after a patient's discharge is also covered by this procedure.				
Appropriate products for the cleaning and disinfection of the surfaces, equipment and medical apparatuses are available.				
PPE for the cleaning personnel are available in different sizes.				
Cleaning personnel have been trained on all relevant procedures, e.g. contact times for the different products, the correct use of PPE (included donning and doffing), and self-monitoring of symptoms. They are aware of the procedure to follow if they develop symptoms				
A record of cleaning staff that have cleaned isolation rooms is maintained.				

Section SXIX: Postmortem Care

Postmortem Care	Completed	In-progress	Not Started	Not applicable
A contingency plan has been developed for managing an increased need for postmortem care and disposition of deceased patients.				

An area in the facility that could be used as a temporary morgue has been identified.				
Local plans for expanding morgue capacity have been discussed with local and regional planning contacts.				
Establish an Infection control policy for postmortem care of COVID 19 suspicious cases.				

Section SXX: Essential support services

Essential support services	Completed	In-progress	Not Started	Not applicable
The impact of COVID-19 on hospital food supplies is anticipated; taking proactive measures to ensure the availability of food.				
Ensure the availability of appropriate back-up arrangements for essential life-lines, including water, power, and oxygen.				
Solicit the input of hospital security in identifying potential security constraints and optimizing the control of facility access, essential pharmaceutical stocks, patient flow, traffic, and parking.				

REFERENCES

Centers for Disease Control and Prevention: Comprehensive Hospital Preparedness Checklist for Coronavirus Disease 2019 (COVID-19). https://www.cdc.gov/coronavirus/2019-ncov/hcp/hcp-hospital-checklist.html

World Health Organization: Hospital Readiness Checklist for COVID-19. http://www.euro.who.int/en/health-topics/health-emergencies/coronavirus-covid-19/novel-coronavirus-2019-ncov-technical-guidance/coronavirus-disease-covid-19-outbreak-technical-guidance-europe/hospital-readiness-checklist-for-covid-19

European Center for Disease Prevention and Control. Checklist for hospitals preparing for the reception and care of coronavirus 2019 (COVID-19) patients. https://www.ecdc.europa.eu/en/publications-data/checklist-hospitals-preparing-reception-and-care-coronavirus-2019-covid-19

Supplementary Materials S5: Invitation Script for Hospital Directors and Participation Consent

Invitation script for hospital directors

AUB Social & Behavioral Sciences
INVITATION SCRIPT

Invitation to Participate in a Research Study
This notice is for an AUB-IRB Approved Research Study
for Dr. Carine Sakr at AUB
Principal investigator: Carine Sakr
Email: cs56@aub.edu.lb
It is not an Official Message from AUB

أنا أتواصل معك لأعلمك عن دراستنا الجديدة: "المستشفيات النائية في زمن الكورونا في لبنان."

تقوم بهذه الدراسة مع كل المستشفيات التي هي جزء من مشروع الجامعة الأميركية في بيروت مع الوكالة الأميركية للتنمية الدولية. تهدف هذه الدراسة إلى فهم مستوى الجهوزية والتحديات التي تواجهها المستشفيات النائية في لبنان, بالإضافة إلى تأثير مشروع الجامعة الأميركية في بيروت مع الوكالة الأميركية للتنمية الدولية على جهوزية مستشفاكم. نخطط إلى مقابلة أربعة من عاملي القطاع الصحي من كل مستشفى شريكة: مدير المستشفى, مسؤول مكافحة العدوى, مسؤول التمريض, ورئيس العمال.

يتم التواصل معك لأن موافقتك كمدير المستشفى ضرورية كي نستطيع أن نجري هذه الدراسة في مستشفاكم.

المقابلة تتضمن أسئلة متعلقة بجهوزية مستشفاكم لجائحة الكورونا, تجهيز الوحدة الخاصة بالكورونا, التحديات التي واجهتوها, وتأثير الجائحة على الموظفين وعلى المجتمع المحيط بالمستشفى. إن الوقت المتوقع لإتمام المقابلة يتروح بين 30-45 دقيقة.

إذا وافقت على مشاركة مستشفاكم في هذه الدراسة, ستجري المقابلات خلال زيارة التقييم الخاصة بمشروع الجامعة الأميركية في بيروت مع الوكالة الأميركية للتنمية الدولية, بحيث سيزوركم فريق البحث الذي كان حاضرا في الزيارة السابقة.

إذا كان لديك أي أسئلة إضافية عن هذه الدراسة, يمكنك التواصل مع الدكتورة كارين صقر

(cs56@aub.edu.lb).

شكرا.

Consent to participate in a Research Study

Title: Remote Hospitals in times of COVID-19 in Lebanon

Investigator: Dr. Carine Sakr

Address: Department of Family Medicine

American University of Beirut Medical Center

Cairo Street

Beirut, Lebanon

Phone: (01) 350 000 ext. 7195

You are being asked to participate in a research study conducted at the American University of Beirut (AUB), Please take time to read the following information carefully before you decide whether you want to take part in this study or not. Refusal to participate will involve no loss of benefits; moreover, you can stop your participation at any time following signing this consent without affecting your benefits and relationship with AUB-Medical Center (AUBMC).

1) Purpose and description of the study

The COVID-19 pandemic has revealed challenges in public health preparedness, especially in low and middle-income countries. These challenges are particularly threatening in remote areas due to economic stagnation, and shortage of equipment and health care providers. To our knowledge, the current data available on response to COVID-19 in hospital settings is mostly from developed countries. The paucity of data in developing countries suggests a need for a better understanding of challenges faced by hospitals and their readiness, to inform better decision making. Our current study aims to understand readiness of private remote hospitals in Lebanon to respond to the COVID-19 pandemic, from a multi-stakeholder perspective involving hospital directors, infection control specialists, in addition to the head of nurse and chief of staff.

We have contacted your hospital director and secured their approval to conduct this study. As your hospital is part of the USAID-AUB initiative, we are conducting this study to have a better understanding of the challenges faced by your hospital to respond to COVID-19 pandemic, and the impact the changes that were introduced to the hospital on your hospital staff and the surrounding community. We also aim to assess the impact of our intervention on the level of readiness in your hospital.

If you agree to participate, you will be part of an interview. Participation should take about 30 - 45 minutes. The interview includes general information about you (age, gender, job title, and whether or not you worked with COVID-19 patients), in addition to questions on the readiness of

your hospital to respond to the COVID-19 pandemic, challenges faced by your hospital during this pandemic, the impact of the USAID-AUB initiative on your hospital, and the impact of these challenges and changes on your hospital's staff and the surrounding community. Participation is on a purely voluntary basis. If you do not wish to answer any particular question in the interview, you may say "skip". All data collected are treated as confidential information. Your name or any identifiers are not included in my research analysis without your explicit permission. All data shared with other investigators have no identifiers that could be linked to your personal responses.
I would like to audio record this interview so as to make sure that I remember accurately all the information you provide. I will keep these on the password protected computer of the principal investigator. Only the principal investigator and the co-investigator will have access to the recordings. The interview records will not be shared with any collaborators nor with your hospital administration. Only the aggregated data from the interviews (which will have no identifiers) will be shared. You may still participate in the interview if you do not want to be recorded.

Potential risks

There are no risks associated with your participation, as you will only be taking part of an interview that does not include any sensitive questions. If at any time and for any reason, you would prefer not to answer any questions, feel free not to. If at any time you would like to stop participating, you can tell me. We can take a break, stop and continue at a later date, or stop altogether. You will not be penalized in any way for deciding to stop participation at any time. If you refuse to participate or decide to withdraw your consent at any time, this will not affect your relationship with your employer nor with the American University of Beirut and its medical center.

2) Potential benefit

Your participation is critical to understand the readiness and the challenges faced by remote hospitals in Lebanon during COVID-19, in addition to understanding the impact of the USAID-AUB project on your hospital's readiness. The study will also allow us to suggest recommendations for your hospital and other remote hospitals to follow in similar scenarios in case they arise.

3) Confidentiality

The interview will be audio taped and stored on the principal investigator's password protected computer. Your name will not be revealed unless you consent to. The audiotapes will be the property of the American University of Beirut. Findings obtained will not affect your relationship with your employer nor with the American University of Beirut and its medical center.

Investigator's Statement

I have reviewed, in detail, the informed consent document for this research study with ________ ________________________ (name of participant) the purpose of the study and its risks and benefits. I have answered all the participants' questions clearly. I will inform the participant in case of any changes to the research and in case a decision to end the subject's participation is taken.

______________________________ ____________________

Name of Investigator or designee **Signature**

____

Date & Time

Subject Participation

I have read and understood all aspects of the research study and all my questions have been answered. I voluntarily agree to be a part of this research study and I know that I can contact

Dr. ___Carine Sakr_______________ at 350000 ext 7195______________or any of her designee involved in the study in case of any questions. If I felt that my questions have not been answered, I will contact the Institutional Review Board for human rights at AUB at __5440/5445_________I understand that I am free to withdraw this consent and discontinue participation in this project at any time, even after signing this form, and it will not affect me. I know that I will receive a copy of this signed informed consent.

__________________________________ ____________________________

Name of Participant **Signature**

____

Date & Time

___________________________________ ____________________________

Witness's Name **Signature**

____

Date

Consent to Record Interview
(Question should be posed at the start of the recording)
Do you also voluntarily consent to this interview being recorded?

- o Yes

- No

Consent to Quote from Interview

I may wish to quote from this interview either in the presentations or articles resulting from this work. A made-up name will be used in order to protect your identity, unless you specifically request that you be identified by your true name.

Do you agree to allow me to quote from this interview?

- Yes
- No

[If you agree to have your name used, please sign below to confirm your choices:

Consent for recording of interview: ___________________________

Consent for quoting from interview: _________________________

Date: ________________________

If you chose to be interviewed and have your name used, you will be given a copy of this consent form with your signatures.]

Supplementary Materials S6: Interview Guide

Interview Guide

Before the start of the interview, I would like to remind you of the following. The purpose of this interview is to gain a better understanding on the readiness of your hospital to respond to COVID-19 pandemic, the challenges your hospital faced during the preparation phase, and how these challenges and changes affected your hospital's staff and the surrounding community. We also aim to understand the impact of the USAID-AUB intervention on your hospital's readiness to the pandemic. This interview will take around 30-45 minutes of your time and will be audiotaped. Your identity will remain anonymous. If you wish to stop the interview at any time, please let me know. You have the right to withdraw your consent at any time with no penalty, and you also have the right to refuse to answer any question. Refusal to answer questions or to withdraw your consent will not affect your relationship with your employer nor with AUB and its medical center.

I. General Information

1. Gender:

- Male
- Female
- Prefer not to answer

2. **Age:**
3. **Job Title:**
   - Hospital Director
   - Infection Control Manager
   - Head of nurses
4. **Which resources (guidelines, etc…) did your hospital use while preparing for the COVID-19 pandemic?**
   - Center for Disease Control and Prevention (CDC)
   - World Health Organization (WHO)
   - Lebanese Ministry of Public Health (MoPH)
   - Other

**Topic**	**Questions**	**Probing Questions**
**Readiness to COVID-19**	How would you describe your hospital's preparedness today to host covid-19 patients?	Why do you think your hospital is ready/ not ready? Can you elaborate?
**Preparation to COVID-19**	When did you start preparing your hospital to host COVID-19 patients? Which steps did you follow to prepare your hospital?	What changes did you introduce to your hospital since the beginning of the COVID-19 pandemic in Lebanon?<br>Give me examples of changes you introduced.

	Do you think the USAID-AUB initiative was helpful in the preparation to respond to the pandemic? How so?	Tell me more about your COVID-19 unit.
**Effect on hospital and the community**	How do you think these changes affected your hospital? (physical setting, new policies, admitting COVID patients)	What kind of costs were incurred on your hospital? How did these changes affect your hospitals' staff? Can you give me examples?<br><br>Do you think you need financial or technical

	What type of support do you think your hospital needs at this stage?<br><br>What do you think of the community surrounding the hospital?	support? Do you need more trainings?<br><br>Were these changes received well by the community?<br>What was the feedback of the community?
**Challenges**	What kind of challenges did you face while preparing the COVID-19 unit in your hospital?<br><br>How did you overcome these challenges?	What were the challenges pertaining to setting up the COVID unit, training healthcare workers, securing personal protective equipment, and securing costs?<br><br>Did you request any support from external sources? (MOPH, municipalities, NGOs, etc…)

		Did you set any specific policies to address these challenges?
**Questions specific for Infection Control**	How would you describe the role of infection control team in the preparation phase?<br><br>What were the challenges faced by the infection control team?	In other words, what was your role as an infection control manager in the preparation of the hospital to respond to COVID-19?<br>How did that impact your other responsibilities as an infection control officer?<br><br>Did you face any challenges in securing the equipment?<br>Did you face challenges in compliance by the healthcare workers?<br>Can you think of any other challenges?
**Questions specific head of nurses**	How do you think the healthcare workers are adapting to the changes introduced to the hospital secondary to the pandemic?	Was there any change in their performance?<br>What about absenteeism/ sick-leave and turnover?<br>Did many healthcare workers get infected? How

	What kind of support is available for healthcare workers during this period?	many? Did they require hospitalization?<br><br>Are there any mental health support services?<br>Are there any specific policies for staff working with COVID patients?
**Additional Questions**	How many healthcare workers got vaccinated against COVID-19 at your hospital (percent)?<br>What is the situation of COVID-19 pandemic at your hospital post-vaccination?<br>Did you have any cases of influenza infection among healthcare workers this year? How do the numbers compare to last year?	
